# Full characterization of an attosecond pulse generated using an infrared driver

**DOI:** 10.1038/srep26771

**Published:** 2016-05-27

**Authors:** Chunmei Zhang, Graham G. Brown, Kyung Taec Kim, D. M. Villeneuve, P. B. Corkum

**Affiliations:** 1 Joint Attosecond Science Laboratory, University of Ottawa and National Research Council of Canada, 100 Sussex Dr, Ottawa K1A 0R6, Canada; 2 Centre for Relativistic Laser Science, Institute for Basic Science (IBS), Gwangju 500-712, South Korea; 3 Department of Physics and Photon Science, Gwangju Institute of Science and Technology (GIST), Gwangju 500-712, South Korea

## Abstract

The physics of attosecond pulse generation requires using infrared driving wavelength to reach the soft X-rays. However, with longer driving wavelength, the harmonic conversion efficiency drops significantly. It makes the conventional attosecond pulse measurement using streaking very difficult due to the low photoionization cross section in the soft X-rays region. *In-situ* measurement was developed for precisely this purpose. We use *in-situ* measurement to characterize, in both space and time, an attosecond pulse produced by ultrafast wavefront rotation of a 1.8 μm fundamental beam. We confirm what models suggest – that each beamlet is an isolated attosecond pulse in the time domain. We get almost constant flat wavefront curvature through the whole photon energy range. The measurement method is scalable to the soft X-ray spectral region.

The nonlinear interaction of an intense ultrashort laser pulse with ionizing matter generates bursts of coherent XUV radiation with attosecond pulse duration^1^. These attosecond pulses open the route to study and control of ultrafast electron dynamics^2^ in attosecond time scale^3^. For most experiments it is important that the pulses are well isolated and, for a range of experiments, it would be useful if the pulses could access the characteristic soft X-ray absorption features of atoms^4^. To reach soft X-ray absorption features, we need infrared drivers^5^,^6^ and we also need methods to characterize pulses that are scalable to the soft X-rays^7^. We report the first temporal characterization of isolated attosecond pulses produced by an infrared driver.

The information for *in-situ* measurement method^8^ is perturbatively – but indelibly – placed onto the attosecond pulse itself. The only previous demonstration of the method was using an 800 nm fundamental beam^8^ and the attosecond pulse was selected by polarization gating^9^.

We show that isolated attosecond pulses can be created with infrared drivers and we measured their spatial-temporal characteristics. In addition the wavefront curvature of each frequency component is characterized and compared with theoretical simulation. We measure that the wavefront curvature weakly depends on the frequency. This changes the space-time structure of the pulse as it propagates^10^,^11^, but the effect is relatively small. At the beam center, the near-field pulse duration is 390 as and it increases to 420 as in the far-field. As suggested previously^12^ in contrast to 800 nm drivers, the long trajectory contribution to the attosecond pulse is small over the whole frequency range. The theoretical reason why the long trajectory contribution is less important is that the difference of the dipole phase between the long and short trajectories becomes much greater for long wavelength driver pulse. Please see Fig. S5. This leads to a large difference in divergence of the radiation from the short and long trajectories. The experimental reason is that, when we optimized for the short trajectory emission, background radiation from long trajectory emission is not observed. The details are discussed in Supplementary Information, Section II.

To create isolated attosecond pulses we choose to use ultrafast wavefront rotation^13^,^14^. Multi-optical cycle laser pulses emit a train of attosecond pulses, separated in time by half an optical period of the driving pulse. Using a fundamental beam with a temporal wavefront rotation spatially separates the attosecond pulses in a train. When viewed through an aperture they are predicted to be isolated attosecond pulses. We generated spatially well-separated attosecond pulses^12^ by gently focusing the infrared beam and using low ionization potential gases. The method can be scaled to stronger focusing and high ionization potential nonlinear media such as Helium. Polarization gating could also be used.

As shown in the schematic diagram of the experimental setup in Fig. 1, wavefront rotation can be induced at the focus by placing a thin wedge in the beam. The amount of rotation is determined by the angle of the wedge and the distance from the wedge to the focusing mirror. For more details, please see Supplementary Information, Section I. When the distance is set to 3.5 meters for a 2.8° wedge, we obtain XUV beamlets that are spatially well-separated in the far-field.

When the wedge is mounted to make the direction of the wavefront rotation parallel to the slit vertically mounted in the XUV spectrometer, each beamlet will pass through the slit at a different height. Figure 2a shows that all beamlets hit the detector. There are three beamlets in the spectrum generated from Kr gas. Each has a continuous spectrum and is spatially distinct. Since the direction of the wavefront rotation is the same as the direction of the spatial modulation induced by the perturbation beam for the *in-situ* measurement, it is preferable to rotate the wedge by 90 degree. This configuration is illustrated in Fig. 1. The slit then selects a single beamlet. The spectrum is shown in Fig. 2b. In either case, the spectrum clearly shows that the divergent emission which is expected from long trajectories is not detectable.

To make sure only one isolated attosecond pulse passes through the center of the slit, we do not only need to set the size of the slit to be less than the spatial separation between two adjacent attosecond pulses, but also to set the carrier envelope phase (CEP) to direct the selected attosecond pulse through the center of the slit.

Figure 2c shows how the spatial profile of the XUV radiation varies with the CEP of the driving laser pulse when the wavefront rotates parallel to the slit. The figure displays the spatial distribution of beamlets, integrated for the whole energy range, (vertical axis) plotted for different values of CEP (horizontal axis). As we scan the CEP, the propagation direction of all beamlets drifts correspondingly due to a time shift of the whole emitted attosecond pulse train under the driving pulse envelope. When the wavefront rotation direction is perpendicular to the slit, as we scan the CEP, the variation of the spatial profile of the XUV radiation is perpendicular to the slit. So the attosecond pulses in the train pass through the slit one after another. Figure 2d shows the intensity plot of the harmonic spectrum as a function of the CEP when the wavefront rotation is perpendicular to the slit. The variation of the harmonic intensity with CEP originates from the different part of the emitted attosecond pulse train selected by the slit.

Next, we added the second harmonic beam at a small angle to the fundamental, perturbing the harmonic generation process in space and time. This perturbation changes the phase of the harmonic radiation and modifies the wavefront of the XUV radiation in near-field. Then the propagation direction and divergence of the XUV emission vary with the time (phase) delay between the fundamental and perturbation laser pulses. In this way, we modulate the far-field spatial and spectral pattern of the beam as we change the phase between two beams.

Figure 3a,b show the measured spectrally-resolved far-field beam profile as a function of the time delay for the photon energy of 30 eV and 60 eV. The vertical axis is the spatial profile of the XUV emission while the horizontal axis consists of 128 images placed side-by-side, each taken at different time delay (each delay step is 140 as).

The far-field distribution contains spatial amplitude and phase information of XUV emission in the near-field and can be determined by using a phase retrieval algorithm^8^. Since the spectra generated with 1.8 μm driving laser only contain the contribution from short trajectory, the phase retrieval algorithm is simplified. Without the quantum path interference^15^ between long and short trajectories, we only need half of the parameters and less time to reconstruct the attosecond pulse. Reconstruction results are shown for the energy of 30 and 60 eV in Fig. 3c,d. The reconstructed results include the spatial amplitude and spatial phase information for the given energy. We apply the phase retrieval algorithm to all energies. Thus, the amplitude and the phase for each XUV frequency are fully reconstructed in space. Then we obtain the relative phase between different frequency components from the oscillation phase of the spatial distribution^16^. The amplitude and phase of the XUV emission are fully determined in space and time.

Figure 4a shows the reconstructed XUV spectrum in the near-field, while Fig. 4b shows the spatial-temporal profile of the pulse. The lower energy of the spectral range was cut by the MCP. Since we used a round MCP, we only took the measured spectrum >27 eV where the spatial profile was not distorted by the edge of the MCP for the reconstruction. Thus, *in-situ* measurement completely reconstructs the attosecond pulse in space and time in near-field where it is produced and, therefore, everywhere as it propagates. Figure 4b shows that the temporal profile of the attosecond pulse does not change very much as we move off the axis. The temporal profiles of attosecond pulse on axis (*θ* = *0*) in near- and far-field are shown in Fig. 4c,d. The characterized pulse is only the radiation lying in the selected spectral window. In practice, a spectral filter would allow us to get the same pulses that we characterize. Since the attosecond pulse is essentially only composed of the radiation from short trajectory electrons this significantly reduces the attosecond pulse duration and gives the whole spectrum a consistent linear atto-chirp of 22 as/eV. The pulse duration in the near- and far-field for *θ* = *0* is about 390 and 420 attoseconds respectively. Please see Supplementary Information, Section III.

In addition, compared to the space-time measurement of the attosecond pulse generated with 800 nm laser pulse^8^, this measurement shows little variation of the temporal profile of the attosecond pulse between on- and off-axis. That is due to the longer driving wavelength, the focusing geometry and the weak long trajectory emission.

Turning to the spatial structure of the beam, in our experiment the gas jet is placed 10 mm (1.5 times the Rayleigh range) before the focus of the fundamental beam. This is much larger than we would typically use for 800 nm experiments, which is 0.8 times the Rayleigh range. The converging wavefront of the fundamental beam is opposite to the intrinsic dipole phase. Consequently, the balance between the fundamental wavefront and the dipole phase makes the harmonic wavefronts nearly flat.

The curves in Fig. 4b correspond to the wavefronts for different photon energies. The 4.5 fs time range on the figure corresponds to about a μm distance, making the horizontal and vertical scales quite different. Therefore, at every frequency the wavefront is almost flat with the curvature flipping near 50 eV photon energy. The measured curvature agrees with the simulation result in the supplementary information file.

In conclusion, we have characterized the spatial and temporal amplitude and phase of isolated attosecond pulses generated by a 1.8 μm pulse. Ours is the first measurement of the spatial properties of an attosecond pulse generated by the lighthouse technique. Often the spatial properties of attosecond pulses are ignored. However, with molecular gases as the nonlinear medium, the spatial structure of a pulse will encode molecular information that complements the spatially averaged spectral information on which high harmonics spectroscopy is currently based. Full characterization of attosecond pulses is, therefore, an important new tool for high harmonic molecular spectroscopy^17^.

We also report the first measurement of the duration of isolated attosecond pulses obtained from mid-infrared driving lasers. This is possible because *in-situ* measurement doesn’t depend on photoelectron spectroscopy but instead the temporal information is encoded on the pulse itself. This encoding allows the method to be extended to any wavelength for which emission is measurable. We measured a 390 as pulse in the medium in which the pulse is generated. With a well characterized, linear chirp (in our case, it is ~22 as/eV), it is now possible to search for the optimum material for chirp compensation^18^. If the chirp were fully compensated, the pulse duration in the far-field would be 210 as while, if the cut-off energy extended beyond 500 eV^19^, we could generate 50 as pulse even without atto-chirp compensation.

## Methods

### Experimental system

In our experiment, 1.8 μm driving laser pulses for creating isolated attosecond pulses are generated from a white-light seeded high-energy optical parametric amplifier (HE-TOPAS, Light Conversion) which is pumped with a chirped-pulse-amplified Ti:sapphire laser system. The OPA system provides 50 fs laser pulses at 1.8 μm with more than 1 mJ pulse energy ~50 fs duration at a repetition rate of 100 Hz. The 1.8 μm pulses are focused into an argon-filled, differentially pumped hollow-core fiber (1 m long, 400 μm inside diameter) by an f = 75 cm lens made of CaF_2_. The spectrum is broadened due to the nonlinear propagation in the hollow-core fiber and the chirp is compensated with an antireflection coated fused silica plate in the beam path^12^,^20^. We routinely achieve ~500 μJ sub-13 fs pulses from the fiber compressor. The CEP of the 1.8 μm laser pulses was measured with an f - 2 f interferometer and locked by a servo system.

In the chamber, the gas source is from a pulsed gas jet with a backing pressure of 4.5 bars. The laser beam is focused by a silver-coated concave mirror (f = 30 cm) into the gas jet. The high harmonics propagate through a slit, reflect from a curved 1200 l/mm grating and are recorded on a microchannel plate and CCD detector. The detector records the spectrum of the XUV emission along its horizontal axis, and the angle of the XUV emission (from the gas jet to the MCP plate) along the vertical axis.

### Isolated attosecond pulse generation

To induce the wavefront rotation into the 1.8 μm laser pulses, we use a thin 2.8° BK7 wedge located in the beam path. Then the laser beam is focused by a silver-coated concave mirror behind the gas jet. The maximum laser intensity at focus is approximately 10^15 ^W/cm^2^. The attosecond pulses generated at different time propagate to different direction.

### Space-time measurement

**Figure 1:**
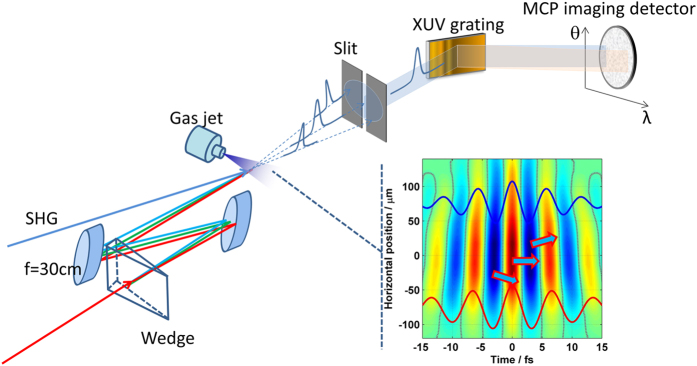
Schematic of the experimental setup. A wedge disperses the pulse prior to the focusing mirror, leading to wavefront rotation. The attosecond pulses generated in each half-cycle of the laser pulse propagate in different directions. A slit selects one attosecond pulse from the spatially separated train. The spectrum of the selected isolated attosecond pulse is measured with the MCP detector. A weak SHG beam is incident at small angle with respect to the fundamental beam. The insert shows the temporal electric-field of the laser pulse at the focus.

For the space-time measurement we use the set-up shown in Fig. 1. The laser beam from the OPA system is divided by a long wavelength pass beam splitter after the second harmonic generation using by a 250 μm -thick BBO crystal. The transmitted beam is used for the isolated attosecond pulse generation. This beam creates the spatially well-separated XUV radiation beamlets extending from 30 eV to 68 eV. The reflected second harmonic is used as a perturbation. Two beams are combined to be parallel but the perturbation beam is 2 mm below the fundamental. Both beams are focused into the chamber. Thus, the angle of the perturbation beam is 6.7 mrad and its intensity was 10-3 of the fundamental. Because of the small angle, the perturbation beam only modifies the wavefront along the vertical direction. The time delay between two pulses is controlled by a piezo stage. The XUV emissions are obtained in Kr gas jet.

## Additional Information

**How to cite this article**: Zhang, C. *et al.* Full characterization of an attosecond pulse generated using an infrared driver. *Sci. Rep.* **6**, 26771; doi: 10.1038/srep26771 (2016).

## Supplementary Material

Supplementary Information

## Figures and Tables

**Figure 2:**
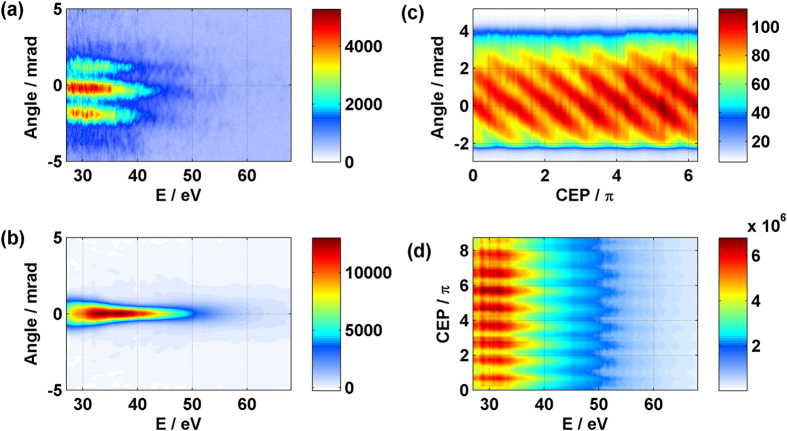
Spectra generated from Kr with sub-two-cycle pulses. (**a**,**b**) The angularly resolved XUV spectrum where the wavefront rotation is parallel (**a**) to the slit and perpendicular (**b**) to the slit. The angularly resolved XUV signals from the left panels are plotted in the right panels as a function of the carrier-envelope phase of the driving laser pulse. (**c**) The drift of the spatial profile due to the time shift of the emitted attosecond pulse train. (**d**) The variation of the harmonic intensity with CEP originating from the selected fraction of the attosecond pulse train passing through the slit.

**Figure 3:**
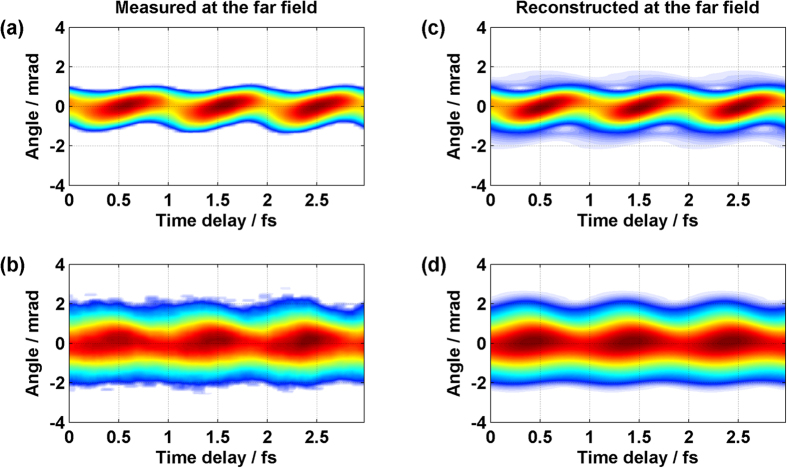
Reconstructing the far-field pattern of an isolated attosecond pulse. (**a**,**b**) Far-field pattern measured as a function of time delay for photon energy of 30 eV (**a**) and 60 eV (**b**). (**c**,**d**) The reconstructed far-field pattern obtained by using the phase retrieval algorithm for the photon energy of 30 eV (**c**) and 60 eV (**d**).

**Figure 4:**
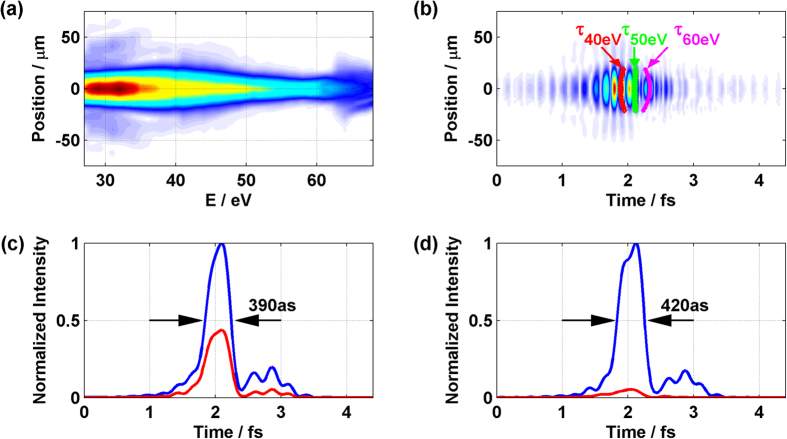
Reconstructed spatial-temporal profile of an isolated attosecond pulse. (**a**) Reconstructed XUV spectrum in the near-field where the XUV is generated. (**b**) The reconstructed spatial-temporal electron field of the attosecond pulse in near-field. The curves (white, green and yellow) correspond to the wavefronts for 40 eV, 50 eV and 60 eV photon energies. (**c**) The intensity profiles of the XUV emission in the near-field for *y* = *0* (blue) and *y* = *10.5 μm* (red). (**d**) The intensity profiles of the XUV emission in the far-field for *θ* = *0* (blue) and *θ* = *0.8 mrad* (red).
