# Supplementary material for: Full characterization of an attosecond pulse generated using an infrared driver
Source: Sci Rep. 2016 May 27;6:26771. doi: 10.1038/srep26771 (PMC4882529; doi:10.1038/srep26771)
Supplement: Supplementary Information [file srep26771-s1.doc]

Supplementary Information on Full characterization of an attosecond pulse generated using an infrared driver

**Chunmei Zhang^1^, Graham G. Brown^1^, Kyung Taec Kim^2,3^, D.M. Villeneuve^1^, P. B. Corkum^1^**

^1^Joint Attosecond Science Laboratory, University of Ottawa and National Research Council of Canada, 100 Sussex Dr, Ottawa K1A 0R6, Canada

^2^Centre for Relativistic Laser Science, Institute for Basic Science (IBS), Gwangju 500-712, South Korea

^3^Department of Physics and Photon Science, Gwangju Institute of Science and Technology (GIST), Gwangju 500-712, South Korea

**I. WAVEFRONT OPTIMIZATION FOR ISOLATED ATTOSECOND PULSE GENERATION**

To induce the wavefront rotation in the 1.8 µm beam, we use a thin 2.8° BK7 wedge located in the beam path as shown in Fig. S1. The wedge adds a slightly different propagation angle to each color, imprinting a linear spatial chirp on the laser beam at the focus.


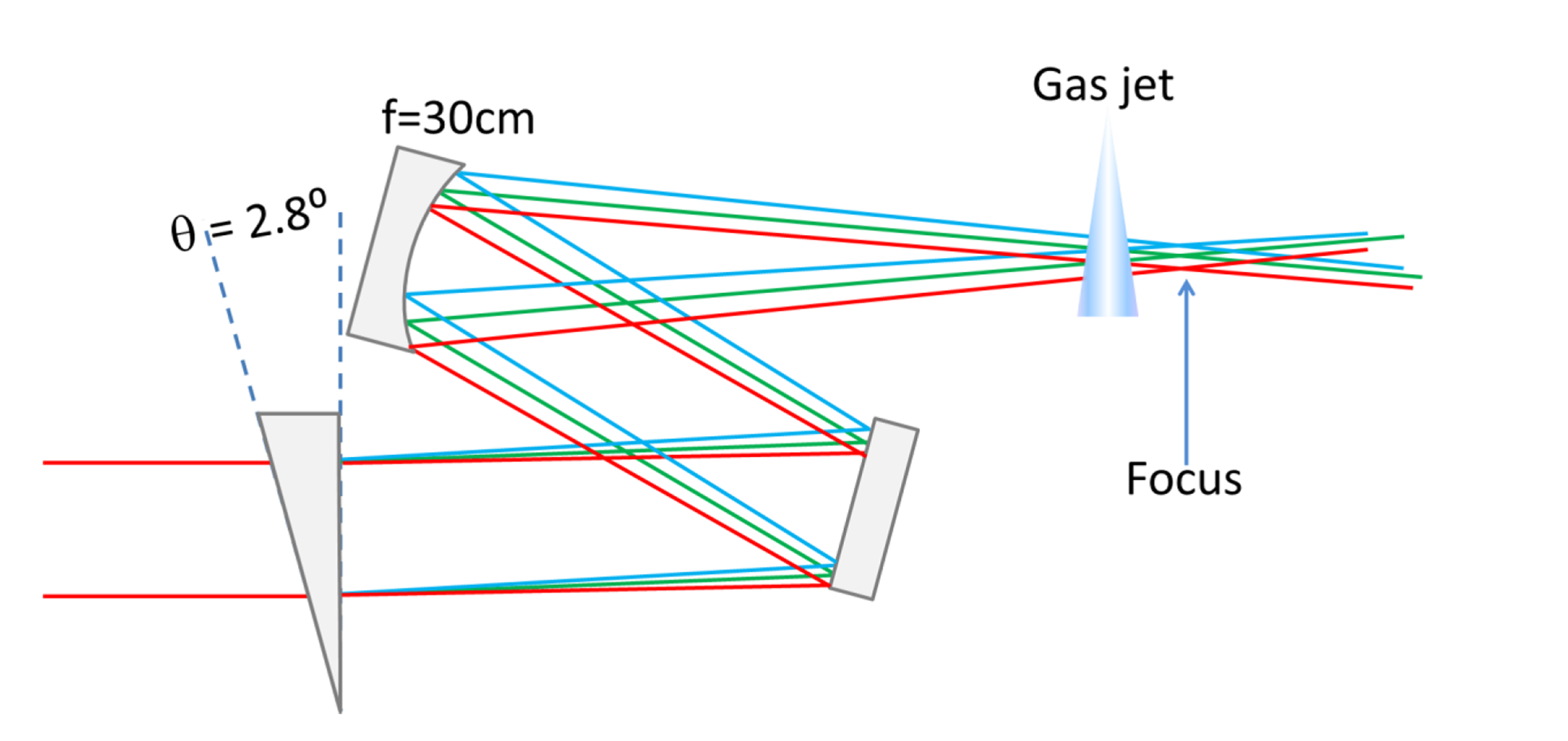


**Figure S1** **Diagram of the focusing 1.8 µm laser with a spatial chirp induced by a thin wedge.**

Here we discuss the optimization of the wavefront rotation (spatial chirp) to get spatially well-separated XUV beamlets.

There are three parameters for optimizing the spatial chirp: the angle of the wedge, the distance between the wedge and the focusing mirror and the jet position. Obviously the spectrum is spread over a larger area as the wedge angle increases. Here we will focus on the other two parameters.

Figure S2 shows the spectrum of the spatially chirped beam measured as a function of vertical position at the focus. The circles indicate the measured center frequency of each spatial position. We use the slope of this plot (wavelength gradient) to characterize the spatial chirp [1].


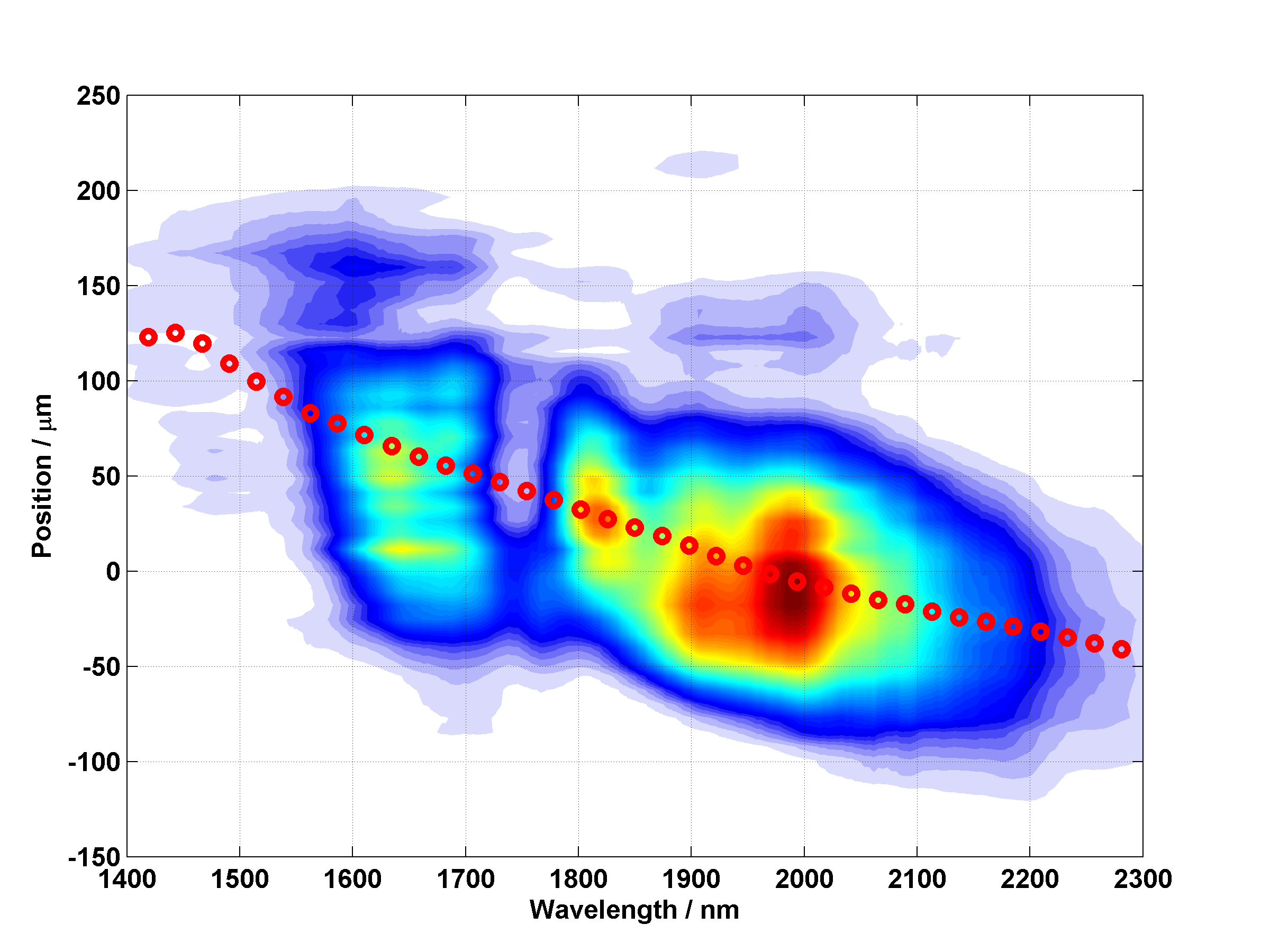


**Figure S2** **Measured spatial-spectral intensity profile of an experimental spatially chirped beam at the focus**. The curve of circles indicates the wavelength gradient.


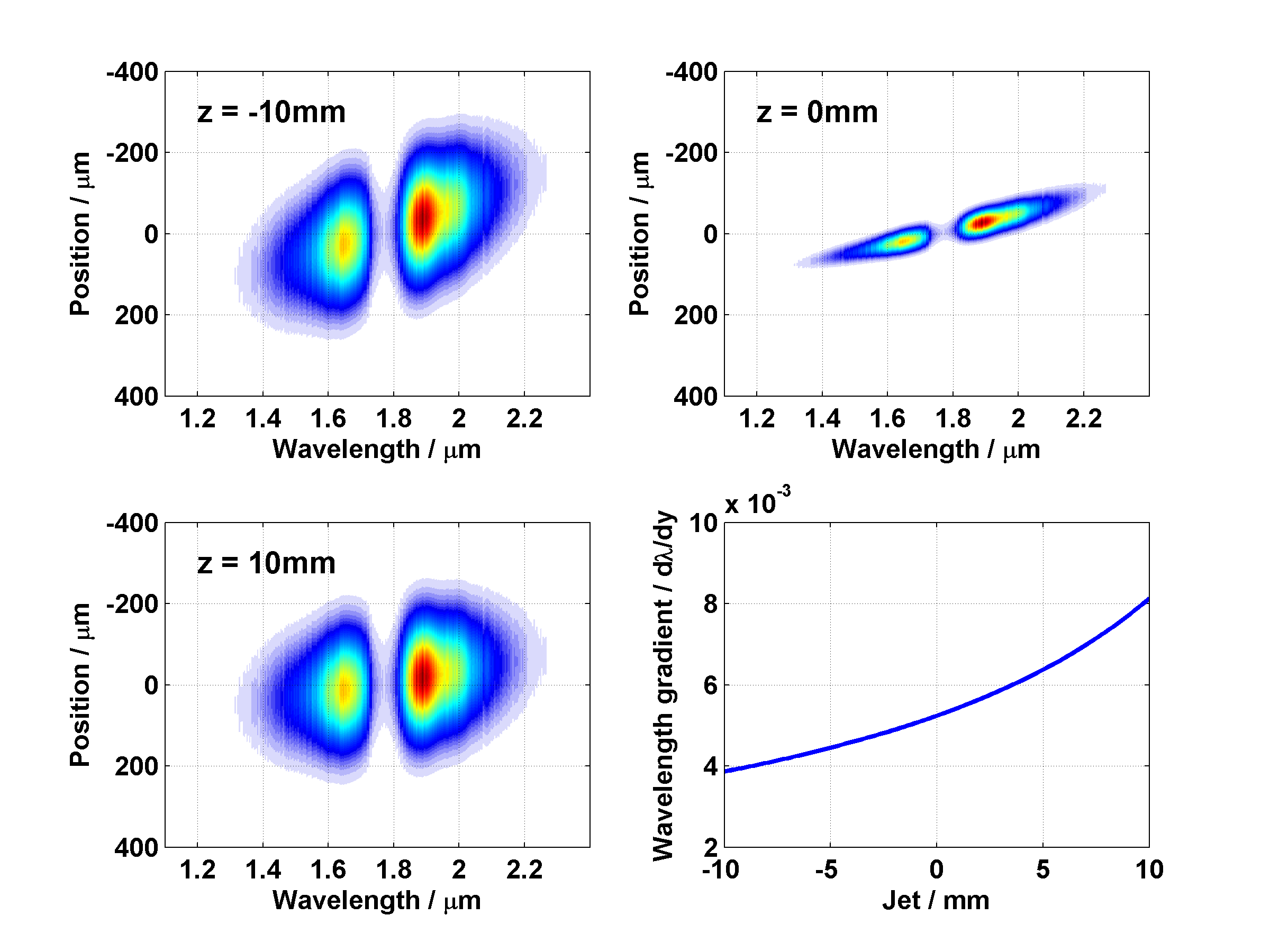


**Figure S3** **Simulated spatial-spectral intensity profile at the gas jet position for -10 mm, 0 mm and 10 mm (before, at and after focus) and the wavelength gradient as a function of the jet position.**

Figure S3 shows the simulated spatial-spectral intensity profile at the gas jet position for -10 mm, 0 mm and 10 mm (before, at and after focus). In the calculation, the beam size was assumed to be 10mm at the focusing mirror. The spatial chirp is induced by a 2.8 degree wedge placing 3.5 m away from the focusing mirror.

The intensity profile for -10 mm shows a larger tilt than that for 10 mm because the prism is imaged after the focus. In Fig. 3d, the wavelength gradient for central wavelength 1.8 µm is plotted out as a function of the jet position. The curve shows the spatial chirp continuously changes as we scan the jet position along the laser propagation direction.


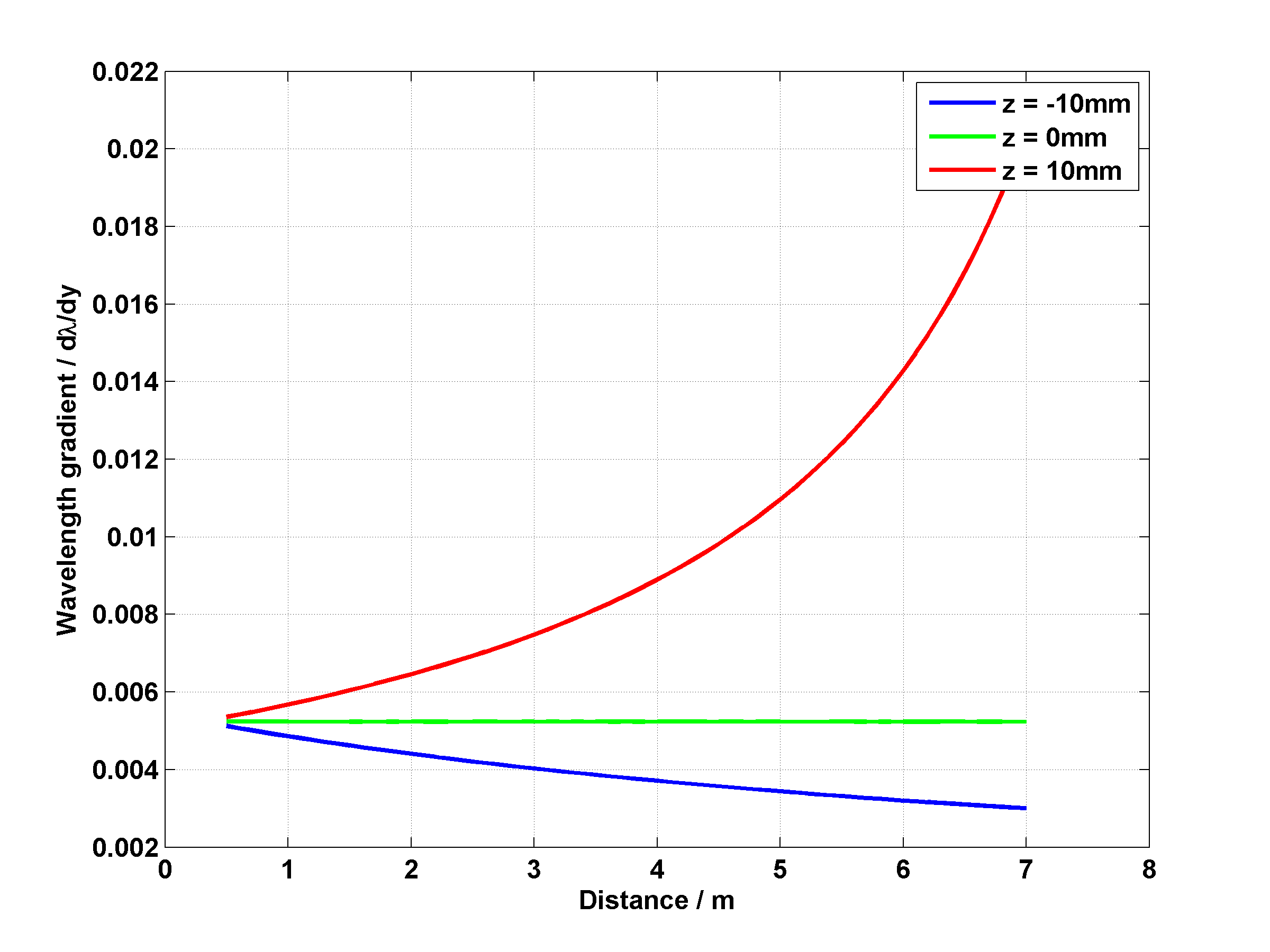


**Figure S4** **Simulated wavelength gradient as a function of the distance between the wedge and the focusing mirror for gas jet position at -10 mm, 0 mm and 10 mm.**

The wedge position also played an important role for the wavefront rotation. Figure S4 shows the calculated wavelength gradient as a function of the distance between the wedge and the focusing mirror for gas jet positions of: -10 mm, 0 mm and 10 mm. At the focus, the wavelength gradient is independent of the wedge position. Before the focus, the wavelength gradient decreases with farther distance, while it is opposite after the focus.

For the experiment, we placed the jet before the focus to reduce the divergence of the beamlets.

**II. ATTO-CHIRP SPATIAL IMPLICATION**

The proportionality factor α relates the harmonic dipole phase to the intensity:

(S1)

where $\omega$ is the angular frequency of the laser field, and are the laser phases at the time of ionization and recombination respectively, $\Phi_{q}$ is the dipole phase of the q^th^ order harmonic and I is the laser intensity.

α depends on the time the recollision electron spends in the continuum before recombination [2], which is different for each harmonic order and trajectory. In general, for a given photon energy, the long trajectory has a much larger α than the short trajectory. In Fig. S5, we plot α for Kr gas as a function of the emitted photon energy for 800 nm (blue) and 1.8 µm (green). In each curve the part with negative tangent is for short trajectory and that with positive tangent is for long trajectory. The curves show that only in the cutoff region, the two trajectories merge into a single one, corresponding to the emission of the highest energy photons.

Equation S1 clearly shows that this factor is proportional to the cubic of driving laser wavelength. Accordingly, the dipole phase difference between long and short trajectories for each emitted energy is relative to the cubic of driving laser wavelength. From Fig. S5, we can see with longer driving laser wavelength, the proportionality factor α for each trajectory becomes greater. In addition, the dipole phase changes rapidly with the intensity which varies spatially. Therefore, the difference of the dipole phase between different spatial portions of the beam also becomes greater resulting in a greater contrast between the short and long trajectories. As we optimize for the short trajectory emission, the long trajectory component from infrared driving would be very dim comparing with that from shorter driving wavelength (800 nm). In experiment, the long trajectory emission is not observed.


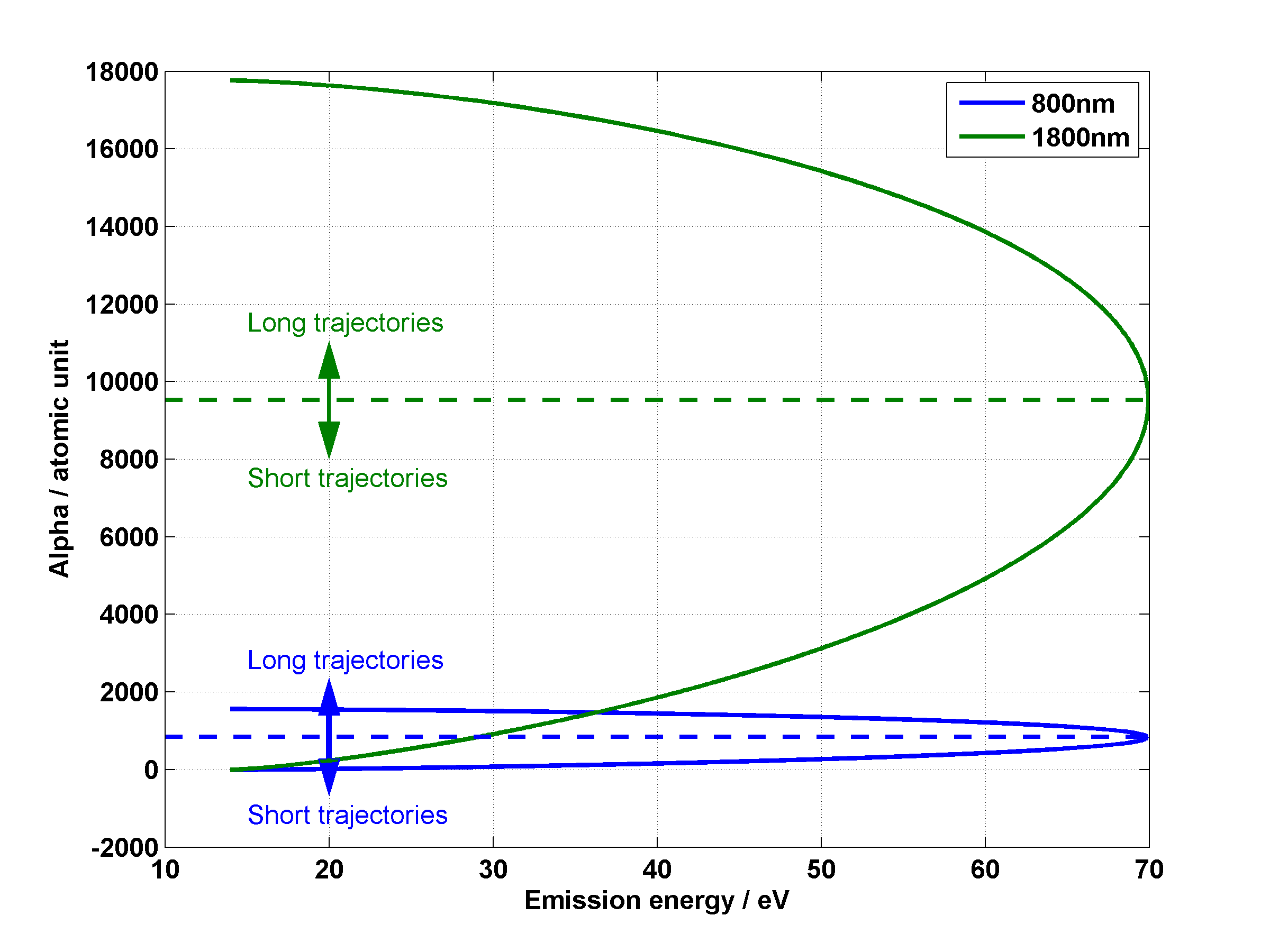


**Figure S5** **Simulated proportionality factor α for Kr gas as a function of the emitted photon energy for long and short trajectory emissions with driving wavelength of 800 nm (blue) and 1.8 µm (green).**

**III. ATTO-CHIRP TEMPORAL IMPLICATION**


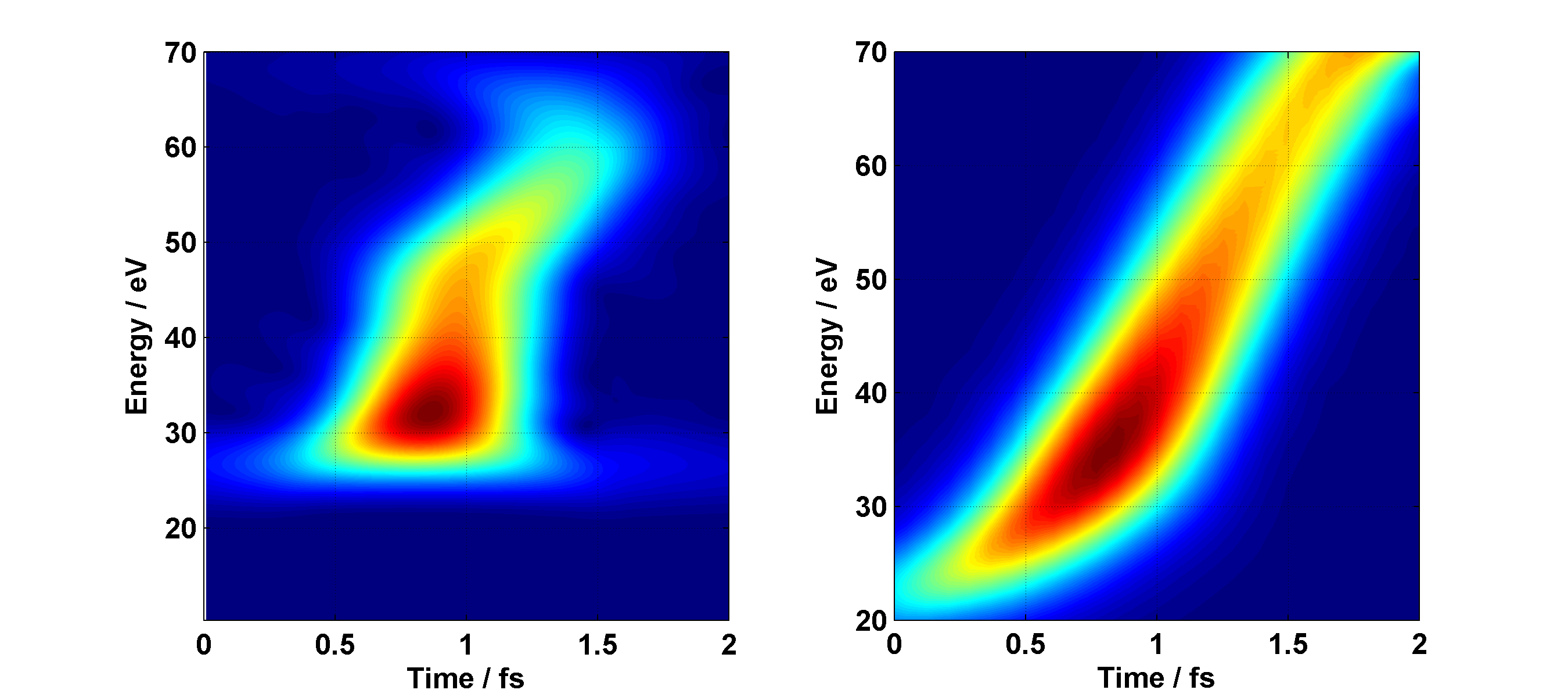


**Figure S6** **Emission time of different frequency components of the attosecond pulse.** Left panel: The experimental measured spectral-temporal profile of the on-axis XUV fields (with atto-chirp ~ 22 as/eV) in the near-field is obtained from the reconstructed amplitude and phase. Right panel: Theoretical result for the emission time of different frequency components of an attosecond pulse (with atto-chirp ~ 24 as/eV) calculated for the single atom response using the strong field approximation. Simulation parameter: laser intensity in the medium 0.6×10^14^ W/cm^2^.

The experimental measured emission time of different frequency components of the attosecond pulses can be seen in the time-frequency plots in the left panel of Fig. S6. The slope of the emission times shows the atto-chirp. The right panel of Fig. S6 shows theoretical calculation for the emission time of different frequency components of the attosecond pulse in the single atom response using strong field approximation. Comparing the measured attosecond pulse to theory, the slope of the emission times agrees with the simulation result. The absence of signal above 65 eV in the experimental result is due to the photo-recombination cross section of Kr atom and the absence lower than 27 eV in the experimental result is due to the MCP size. The comparison demonstrates that the spectral phase is not distorted by the ultrafast wavefront rotation and agrees well with the single atom response within the strong field approximation.

**IV. FREQUENCY DEPENDENT WAVEFRONT CURVATURE**


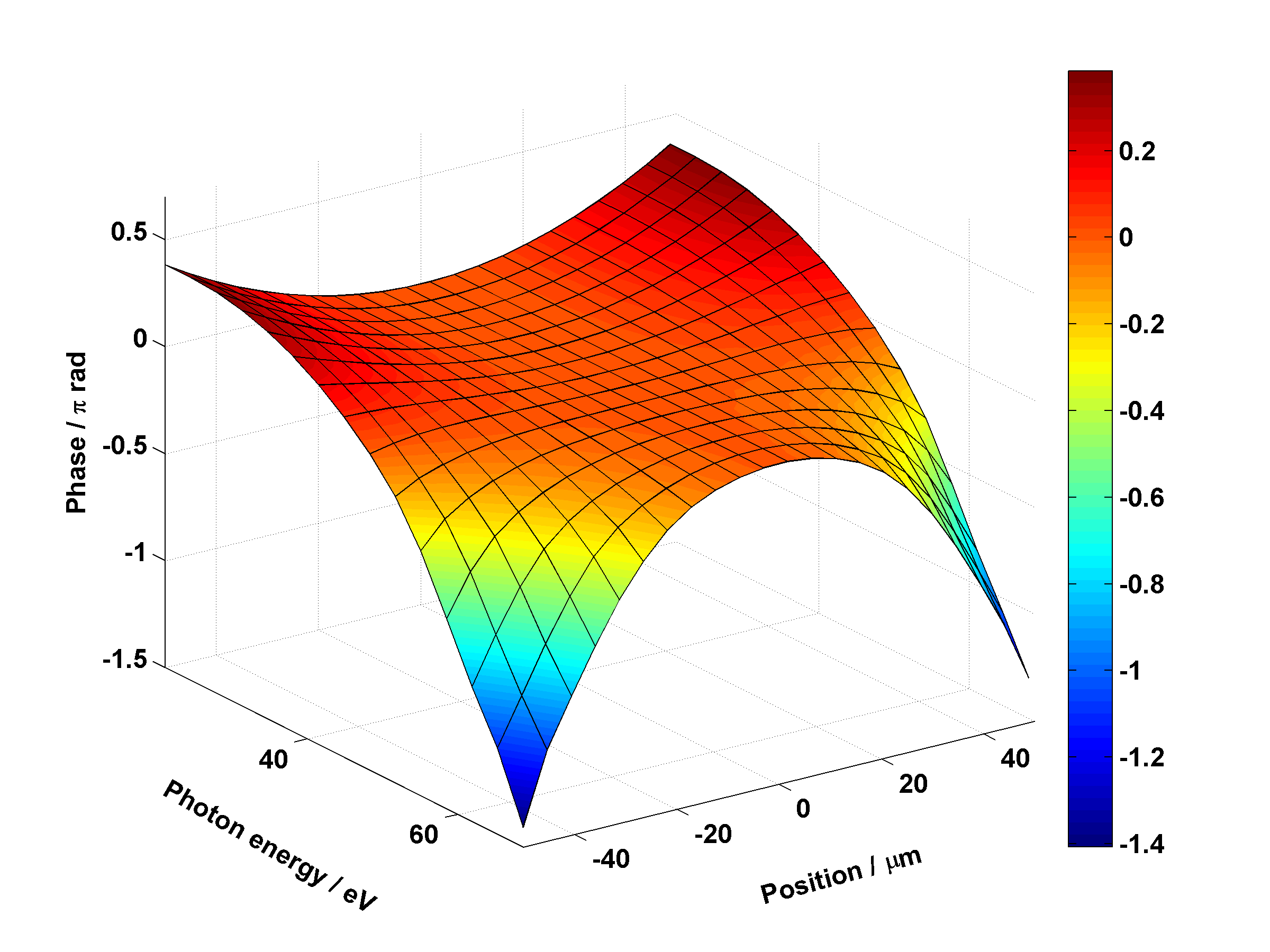

**Figure S7** **Theoretical calculated parabolic structure of the harmonics as a function of harmonic photon energy**.

We have simulated the wavefront curvature [3] for different XUV frequencies. The simulation parameters were a 13 fs pulse, 70 µm beam waist (corresponding to a Rayleigh range ~8.5 mm), with the gas jet placed 10 mm before the focus of the fundamental, and a peak intensity of 0.75×10^14^ W/cm^2^. The model assumes a radially symmetric beam. Consequently, we plot the parabolic structure the harmonics as a function of harmonic order shown in Fig. S7. The wavefronts are relatively flat, and the lower and higher portions of the energy spectrum have opposite curvature. The flat wave front is ~51 eV. It is agreed with our experimental result.

By placing the jet before the focusing of the beam, the converging wavefront phase of the fundamental counteracts the dipole phase. Consequently, the harmonic wavefronts are nearly flat.

**V. UNCERTAINTY OF PHASE RECONSTRUCTION**

Figure S8 shows the calculated the root-mean-square deviation of the reconstructed phase for each energy component. We obtain ΔΦ < 0.08rad within spectral range from 37 to 70 eV, as shown in the following figure. As the curve shows, the phase uncertainty is greater for low photo energy.

#
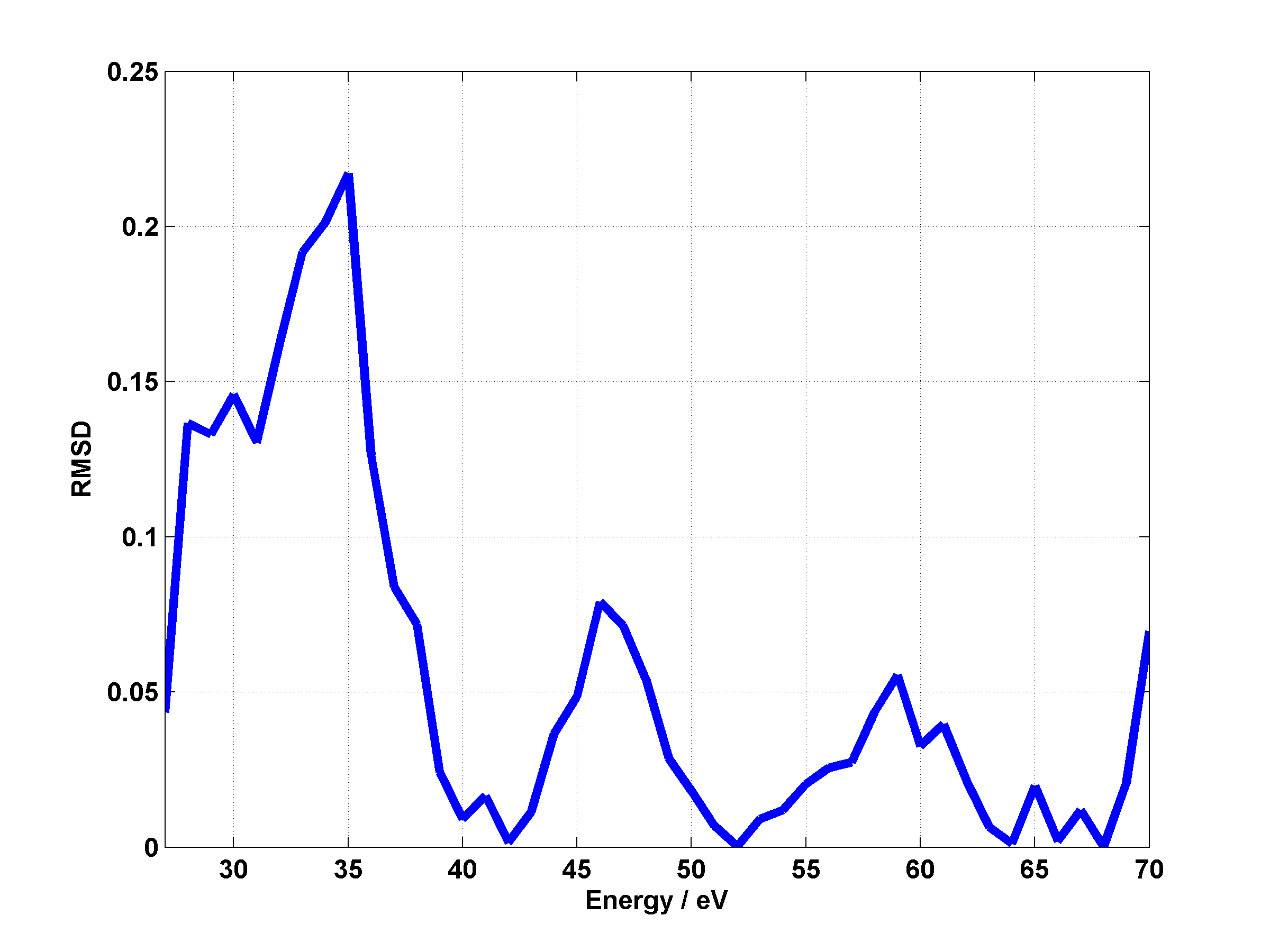


**Figure S8** **Calculated the RMSD for the phase reconstruction of each energy component.**

**References**

[1] Gu, X., Akturk, S. and Trebino, R. Spatial chirp in ultrafast optics” *Opt. Communications* **242**, 599–604 (2004).

[2] Le, A. T. at al. Quantitative rescattering theory for high-order harmonic generation from molecules. *Phys. Rev. A* **80**, 013401 (2009).

[3] Frumker, E. et al. Order-dependent structure of high harmonic wavefronts. *Opt. Express* **20**, 13870-13877 (2012).
